# Suggested Safe Harbor Limit for Titanium Dioxide: An Exposure Level Which Protects Consumers from Cancer Incidence

**DOI:** 10.3389/fonc.2015.00076

**Published:** 2015-03-26

**Authors:** Masood A. Shammas, Dildar Ahmad, Minh D. Nguyen, Samiyah Rajput, Jim Unmack, Gulzar Ahmad

**Affiliations:** ^1^Department of Adult Oncology, Harvard (Dana Farber) Cancer Institute, VA Health Care System, Boston, MA, USA; ^2^InfoTox International Inc., Riverside, CA, USA; ^3^Gnosis International, LLC., Garden Grove, CA, USA; ^4^California State University, San Bernardino, CA, USA; ^5^Unmack Corporation, San Pedro, CA, USA

**Keywords:** titanium dioxide, cancer, California Prop-65, safe harbor limit, OSHA, permissible exposure limit, warning label, cosmetics

Exposure to harmful chemicals can induce genomic instability leading to oncogenic transformation of human cells. Establishing safe harbor limit (SHL) for potentially carcinogenic chemicals is, therefore, critical to minimize exposure and reduce cancer incidence. Unfortunately, a number of chemicals, which may be potentially carcinogenic, are still without any SHL data, and there is an urgent need to have such information available to public. Here, we present titanium dioxide as an example to discuss this important problem.

Titanium dioxide is one of the top 50 chemicals produced worldwide. It has a variety of uses, ranging from use in paints, in foods, in consumer products, and cosmetics. Titanium dioxide is listed in Regulation (EC) No. 1223/2009 of the European Parliament and the Council of 30 November 2009 on cosmetic products) as colorant (Annex IV; List of colorants in cosmetic products); and as UV filter (Annex VI, UV Filters allowed in cosmetics). It is also permitted by the US Food and Drug Administration (FDA) for use in cosmetics generally, including eye area. Similarly, it is NOT listed in the Canadian Ingredient Hotlist.

Based on the LD50s titanium dioxide can be considered as an inert and non-toxic substance. The LD50 (oral, rat) is reported to be over 10,000 mg/kg body weight (1) and the LD50 (dermal, Hamster) is reported to be ≥10,000 mg/kg body weight (2). As far as cancer is concerned, according to IARC, there is inadequate evidence in humans for the carcinogenicity of titanium dioxide. However, there is sufficient evidence in experimental animals for the carcinogenicity of titanium dioxide. IARC’s overall evaluation considered titanium dioxide as a possible carcinogen to humans (Group 2B) (3). On the other hand, American Conference of Governmental Industrial Hygienists (ACGIH) considers titanium dioxide as a non-classifiable human carcinogen (4).

On September 2, 2011, the California Office of Environmental Health Hazard Assessment (OEHHA), listed titanium dioxide (airborne, unbound particles of respirable size) as a cancer causing agent (through inhalation), under California Safe Drinking Water and Toxic Enforcement Act of 1986 commonly known as California proposition 65. State of California EPA (OEHHA), Safe Drinking Water and Toxic Enforcement Act of 1986 (5).

According to the California Safe Drinking Water and Toxic Enforcement Act of 1986 (SDWTEA) (California health and safety code section 25249.6), as of September 2, 2012, no person in the course of doing business shall knowingly and intentionally expose an individual to titanium dioxide without first providing a clear and reasonable warning.

California’s Safe Drinking Water and Toxic Enforcement Act (SDWTEA) requires that exposure assessments be done on items, which may release chemicals known to the State of California to cause cancer and/or reproductive harm and if the assessed exposures exceed the SHLs (No significant risk levels or NSRLs for carcinogen and/or the maximum allowed dose levels or MADLs for reproductive toxins), established by the OEHHA, State of California, then appropriate warning labels must be applied onto the consumer product.

As no SHL has so far been published by the State of California, the manufacturers are at loss as to what level of titanium dioxide they should use in their products, where the consumers are not only protected from exposure to titanium dioxide from their products but also remain in compliance with the SDWTEA, without having to apply the warning labels to their products.

To meet the pressing and urgent need of certain industry, especially that of cosmetics, we propose a mathematical model to derive SHL for titanium dioxide based on the California division of occupational safety and health (Cal/OSHA) permissible exposure limit (PEL), which was derived from the ACGIH threshold limit value (TLV).

The PEL of titanium dioxide is 10 mg/m^3^ for airborne particles excluding nanoparticles. Although nanoparticles are generally defined as having at least one dimension (length, width, or diameter) <100 nm, the PEL is generally applied to particles larger than 5 μm. Therefore, for particles larger than 5 μm, the SHL could be derived from the PEL by allowing for 24 h exposure per day, instead of occupational exposure of 8 h/day, 7 days/week instead of five working days per week, 70 years of life span instead of 40 years of working, assuming inhaling 10 m^3^ during work hours and 10 m^3^ during non-work hours each day. Applying these modifiers to the PEL of 10 mg/m^3^ yield a level of 2 mg/m^3^ for an environmental exposure [10 mg/m^3^ × (8 h/day/24 h/day) × (5 day/week/7 days/week) × (40 years/life/70 years/life) = 2 mg/m^3^]. Using an uncertainty factor (UF) of 3 for the variability of the general population yields a value of 0.7 mg/m^3^ (2 mg/m^3^ ÷ 3 = 0.7 mg/m^3^). To achieve a SHL for titanium dioxide particles larger than 5 μm, another UF of 2 is applied to 0.7 mg/m^3^ and the resultant is multiplied with 20 m^3^/day to get the SHL (UF of 2 is used for materials in common usage where no adverse effects are reported). This value comes out to be 7 mg/day for the general public (0.7 mg/m^3^ ÷ 2 × 20 m^3^/day = 7 mg/day). We used a life span of 70 years for this model because United States Environmental Protection Agency (EPA) assumes expected life expectancy of 70 years for conducting risk assessment on various chemicals including pesticides (EPA, Staff Background Paper # 4, TRAC 5/27/98, updated May 22, 1998 http://www.epa.gov/pesticides/trac/).

We like to make it clear that the SHL mentioned above has no legal value and therefore, we do not recommend or suggest the use of this value unless it is approved and supported by the applicable regulators (CalEPA). However, the validation of this value in experimental model and assay systems can be done to expedite the process of establishing actual SHL for titanium dioxide. This will not only help the titanium dioxide manufacturing industry but will also help the industry using titanium dioxide in their products such as that of cosmetics.

## Conflict of Interest Statement

The authors declare that the research was conducted in the absence of any commercial or financial relationships that could be construed as a potential conflict of interest.
